# Pearson syndrome: a multisystem mitochondrial disease with bone marrow failure

**DOI:** 10.1186/s13023-022-02538-9

**Published:** 2022-10-17

**Authors:** Ayami Yoshimi, Kaori Ishikawa, Charlotte Niemeyer, Sarah C. Grünert

**Affiliations:** 1 Department of Pediatric Hematology and Oncology, Medical Center, Faculty of Medicine, University of Freiburg, Freiburg, Germany; 2 Faculty of Life and Environmental Sciences and Graduate School of Life and Environmental Sciences, University of Tsukuba, Tsukuba, Ibaraki Japan; 3 Department of General Pediatrics, Adolescent Medicine and Neonatology, Faculty of Medicine, University Medical Center, University of Freiburg, Freiburg, Germany

**Keywords:** Pearson syndrome, Natural history, Mitochondrial DNA deletion

## Abstract

Pearson syndrome (PS) is a rare fatal mitochondrial disorder caused by single large-scale mitochondrial DNA deletions (SLSMDs). Most patients present with anemia in infancy. Bone marrow cytology with vacuolization in erythroid and myeloid precursors and ring-sideroblasts guides to the correct diagnosis, which is established by detection of SLSMDs. Non hematological symptoms suggesting a mitochondrial disease are often lacking at initial presentation, thus PS is an important differential diagnosis in isolated hypogenerative anemia in infancy. Spontaneous resolution of anemia occurs in two-third of patients at the age of 1–3 years, while multisystem non-hematological complications such as failure to thrive, muscle hypotonia, exocrine pancreas insufficiency, renal tubulopathy and cardiac dysfunction develop during the clinical course. Some patients with PS experience a phenotypical change to Kearns-Sayre syndrome. In the absence of curative therapy, the prognosis of patients with PS is dismal. Most patients die of acute lactic acidosis and multi-organ failure in early childhood. There is a great need for the development of novel therapies to alter the natural history of patients with PS.

## Background

Pearson Syndrome (PS) was first described in 1979 by Howard Pearson as a disorder of refractory sideroblastic anemia with vacuolization of marrow precursors and exocrine pancreatic dysfunction [1]. Nine years later, Rötig et al. discovered that PS is caused by deletion of mitochondrial DNA (mtDNA) [2]. The prevalence of PS is approximately 1: 1,000,000 [3]. To date, less than 150 cases have been reported worldwide [3–7]. PS is a unique primary mitochondrial disease (PMD), which typically presents with severe hypoproliferative anemia in early infancy followed by progressive symptoms and multi-organ dysfunctions including lactic acidosis, pancreatic insufficiency, renal tubulopathy, failure to thrive, muscle hypotonia, and endocrine disorders [1, 3, 4, 6–8]. Interestingly, about two-thirds of patients with PS experience spontaneous resolution of anemia and become transfusion dependent during their clinical course. Therapeutic approaches are so far only symptomatic, and patients with PS have a dismal prognosis. The majority of patients die before 6 years of age [3–5, 7, 8]. Recently, increasing attention is being paid to PS due to development of novel therapies for PMDs, which can potentially be applied in children with this rare disease [9–12].

## Main text

### Mitochondrial disease and mitochondrial DNA deletion syndrome

PMDs are a clinically and genetically heterogeneous group of disorders characterized by dysfunction of the mitochondrial respiratory chain and caused by pathologic variants of genes encoded by either mtDNA or nuclear DNA [13, 14]. mtDNA encodes for 13 polypeptides that are essential subunits of complexes I, III, IV, and V, as well as 22 tRNAs and 2 rRNAs that are necessary for translation of these 13 polypeptides. Each cell in the body obtains hundreds and thousands of copies of mtDNA, depending on the cell type. Many patients with a PMD have a mixture of both wild-type and mutated mtDNA in the same cell, called heteroplasmy. Distribution of mutated mtDNA differs in different tissues, possibly by random partitioning during early cell division. Cellular dysfunction appears only if the level of heteroplasmy exceeds a certain critical threshold (threshold effect).

PS is caused by single large-scale mtDNA deletions (SLSMDs) of variable size (1.5 to 8.0 kb) and location [4–6, 15]. About 40–50% of PS patients carry the “common deletion” with a length of 4977 bp [4, 5, 16]. SLSMDs are also found in children or young adults with chronic progressive external ophthalmoplegia (CPEO) or Kearns-Sayre syndrome (KSS) characterized by PEO, pigmentary retinal degeneration, ataxia and cardiac conduction block. PS, CPEO and KSS form a continuous spectrum of diseases called “mtDNA deletion syndromes” with a common genetic event [4, 17]. Of note, patients with PS surviving early childhood can develop a KSS-like phenotype [3, 15, 18].

### Pathogenic mechanisms and mouse model of mtDNA deletion syndrome

**Fig. 1:**
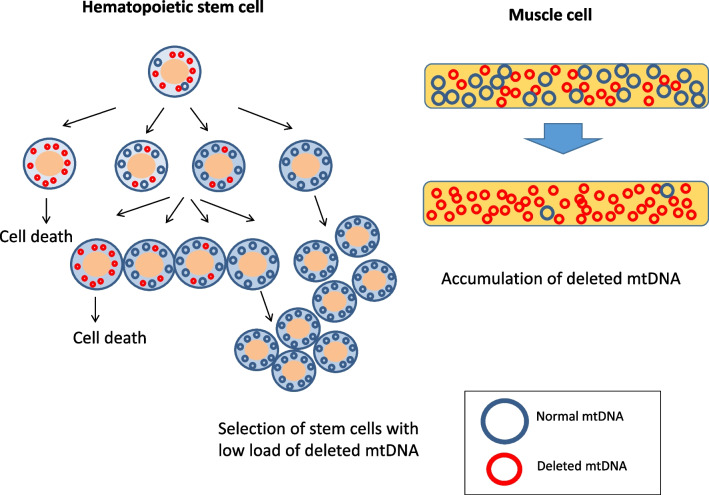
Hypothesis for the phenotypical change in Pearson syndrome. Hematological recovery is hypothesized to be due to a positive selection of hematopoietic stem cells harboring low load of deleted mitochondrial DNA (mtDNA). In contrast, deleted mtDNA accumulates in muscle, resulting in the development of Kearns-Sayre syndrome with muscular complications

The unique clinical course of patients with mtDNA deletion syndromes raises interesting questions about the disease mechanisms: (1) How does the same mtDNA deletion cause diverse disorders (PS, KSS and CPEO)? (2) What is the mechanism of spontaneous resolution of anemia in PS? (3) How and why does the clinical phenotype change from PS to KSS? It has been hypothesized that varied loads of deleted mtDNA in different tissues are the main cause for different disease phenotypes. Patients with PS may have a higher proportion of deleted mtDNA in varied tissues overall than those who present in later life with KSS or CPEO [19]. Hematological recovery in PS may be due to a positive selection of hematopoietic stem cells (HSCs) harboring a low amount of deleted mtDNA (Fig. 1) [15, 20]. In contrast to hematopoietic cells, deleted mtDNA may accumulate in tissues such as muscles with time, resulting in the development of neuro- and muscular complications seen in KSS.

Studies in a mouse model of mtDNA deletion syndrome (mito-miceΔ) carrying various levels of heteroplasmy support the above hypothesis [21, 22]. Neonatal mice with a high proportion of deleted mtDNA (> 50%) develop a PS-like severe clinical phenotype [22]. Some of them die soon after birth, and surviving mice can achieve remission from the PS-like phenotype but develop KSS-like disease later. Mice with a moderate burden of deleted mtDNA (11–49%) are healthy at birth but develop KSS-like disease later, while mice with a low proportion (up to 10%) remain healthy. It was also shown that the proportion of deleted mtDNA in blood and liver decreases with age in this mouse model, while time-dependent accumulation of deleted mtDNA was observed in other tissues including muscle where the amount of deleted mtDNA reaches approximately 70 to 80% in various tissues when the mice develop KSS-like disease.

In PS patients, the proportion of deleted mtDNA in peripheral blood is very high (usually > 70%) at diagnosis [4–6]. A few autopsy reports of patients with PS provided information on heteroplasmy status in various tissues and organs, and revealed a high load of deleted mtDNA of more than 70–80% in all tissues [23–25]. Although some reports have shown that severely affected tissues tend to contain a higher proportion of deleted mtDNA [26], a clear association between the proportion of deleted mtDNA in a specific tissue and clinical severity of tissue-specific symptoms is not always present [23]. Therefore, additional factors such as the energy demand and reserve of different tissues may contribute to the phenotype [23, 25]. Studies in with a small number of PS patients have shown that the amount of deleted mtDNA in blood and bone marrow (BM) cells decreased with improvement of anemia [20, 27]. In contrast, repetitive muscle biopsy specimens in patients with KSS showed that the proportion of deleted mtDNA increased with time from 50 to 60% to more than 80%, paralleling the progression of the disease [15].

### Inheritance

Although the vast majority of PS cases are sporadic and caused by a somatic mutational event during early embryonic development, there are a few reports of mothers with CPEO who had children with PS [28–30]. mtDNA is inherited exclusively from the mother [13]. Therefore, deleted mtDNA can be transmitted from the mother to her children. Only a few copies of maternal mtDNA are transmitted to the offspring (a mitochondrial genetic bottleneck), leading to markedly different levels of heteroplasmy in offspring and mother. The unpredictable heteroplasmy status in a child of a mother with mtDNA deletions renders genetic counselling a challenge. In a large study including 226 families with at least one index patient with a mtDNA deletion syndrome, clinically unaffected mothers are highly unlikely to have more than one affected child, while affected women with CPEO had, on average, a one in 24 risk of having a child affected with a mtDNA deletion syndrome [30].

## Diagnosis of Pearson syndrome

### Clinical presentation

PMDs can affect virtually any tissue and organ, but typically, organs that are highly dependent on aerobic metabolism, such as muscle and brain are involved. PS is a clinically unique PMD, in which anemia is often the sole presenting symptom and other features suggesting PMD are lacking [3, 6, 31]. Other common initial symptoms are failure to thrive and gastrointestinal symptoms such as vomiting, diarrhea and feeding difficulties [7, 31]. In our recently published study on 25 individuals with PS, anemia developed at a median age of 5 months (range 0–31 months) [6]. Pregnancy and birth are usually uneventful [3, 6]. However, about 10% of patients have intrauterine growth restriction [31]. Furthermore, Farruggia et al. reported that 27% of newborns of PS are small for gestational age [3]. Some patients present already in the neonatal period with anemia, lactic acidosis, and/or other organ involvement [3, 6, 8]. In general, clinical symptoms in early infancy are often nonspecific, such as muscular hypotonia, failure to thrive, vomiting and chronic diarrhea. In addition, episodic metabolic crises with lactic acidosis can occur.

### Hematological findings at diagnosis

**Fig. 2:**
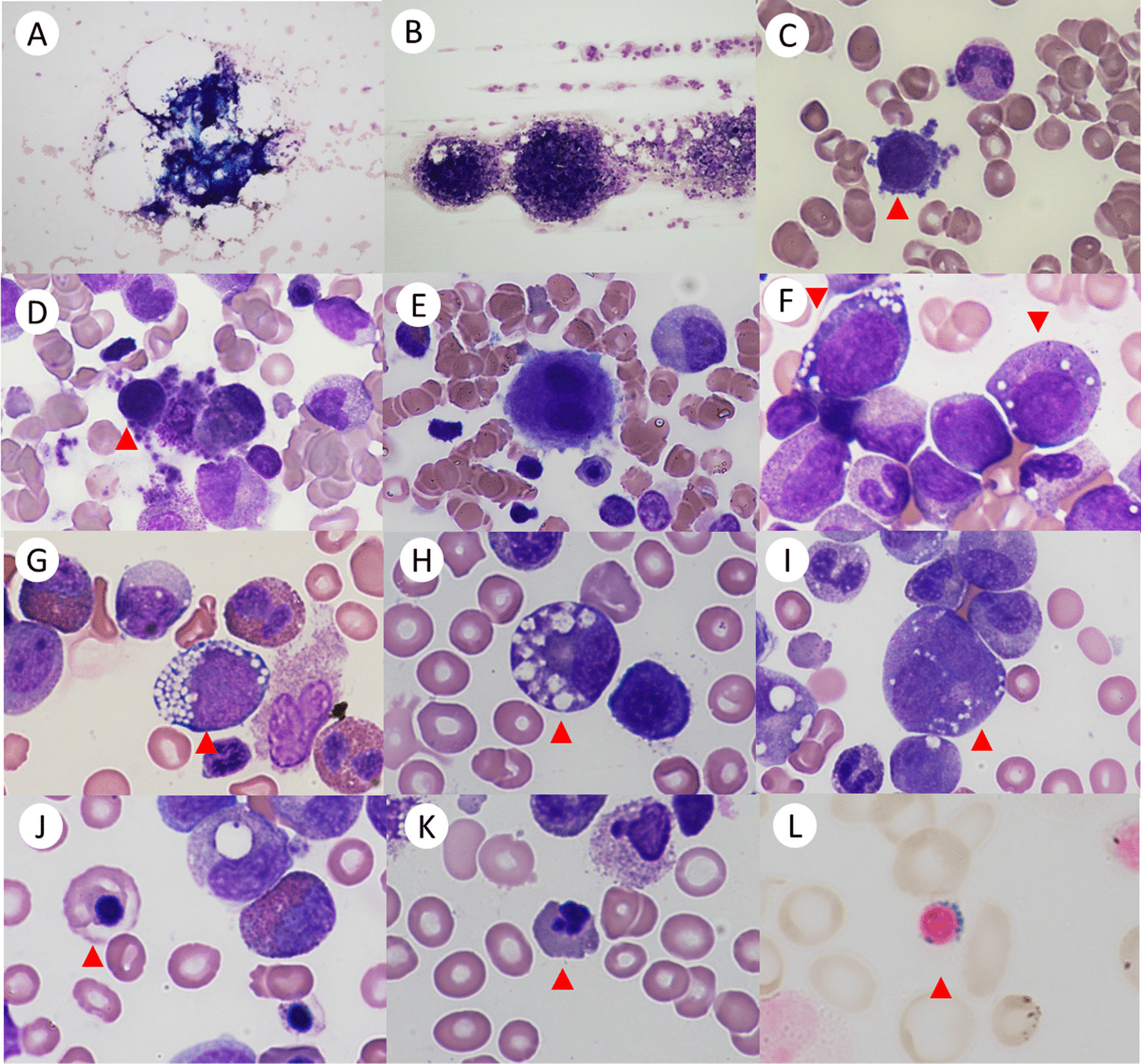
Bone marrow findings in patients with Pearson syndrome. Bone marrow can be hypocellular **A** or normocellular **B**. **C** + **D**: micromegakarocytes, **E**: dysplastic megakaryocyte with bi-nuclei. **F**: proerythroblast and myelocyte with vacuoles. **G**: proerythroblast with vacuoles, **H**: myelocyte with vacuoles, **I**: promyelocyte with vacuoles and double nuclei, **J**: macrocytic normoblast with disturbed hemoglobinization. **K**: erythroblast with lobulated nuclei. **L**: ring sideroblast (iron-staining)

Patients with PS show macrocytic or normocytic hypogenerative anemia [3, 6, 31]. Majority of patients have neutropenia and thrombocytopenia as well [3–6, 32]. In our German/Austrian study with 25 patients, additional neutropenia and thrombocytopenia was found in 80% and 72%, respectively [6]. Rötig et al. reported that 16 of 21 patients (76%) with PS had thrombocytopenia and/or thrombocytopenia [5]. The fetal hemoglobin (HbF) concentration and the level of erythropoietin are elevated in the majority of patients [3, 6]. BM cellularity is typically normal or reduced. Dysplastic features in all cell lineages are common (Fig. 2), and the myeloid lineage often shows disturbed maturation. Vacuolization in myeloid and erythroid precursors is a unique morphological feature of PS detected in nearly all patients [3, 6, 33]. Iron staining reveals ring-sideroblasts in 70–85% of these patients [3, 6]. While detection of ring-sideroblasts is helpful to ensure the diagnosis of PS [3], these typical BM findings can be lacking in the first months of life [33].

### Biochemical findings and other laboratory tests

There are no pathognomonic biomarkers for PS. Like in other PMD, elevated serum lactate concentrations are found in the majority of PS patients [3, 4]. However, in some patients, these abnormalities are only detected intermittently. The ratio of lactate to pyruvate is usually higher than 20 in patients [26]. Organic acid analysis in urine by gas-chromatography mass spectrometry typically reveals elevated concentrations of lactate and the citric cycle intermediates fumarate and malic acid. In some cases elevated excretion of 3-methylglutaconic acid can be observed as a marker of mitochondrial dysfunction [3, 34]. Patients can also display ketonuria with elevated concentrations of 3-hydroxybutyrate in urine [4]. Amino acid analysis shows elevated concentrations of alanine in most patients, which is explained by interruption of the citric cycle resulting in accumulation of pyruvate that can be reversibly converted to lactate or alanine [3, 35]. Additionally, low levels of citrulline and arginine have been reported [35]. Although none of these abnormalities is specific for PS and can also be present in other mitochondriopathies [4], the metabolite pattern can be helpful for the differential diagnosis from other non-mitochondrial BM failure disorders [3]. Carnitine levels can be normal or low in PS and can decrease during the course [36] [37] [38].The diagnostic work-up of a child with PS also includes endocrinological investigations and evaluation of the exocrine pancreatic function to rule out pancreas insufficiency, which is often already present at diagnosis.

### Diagnostic algorithm

**Fig. 3:**
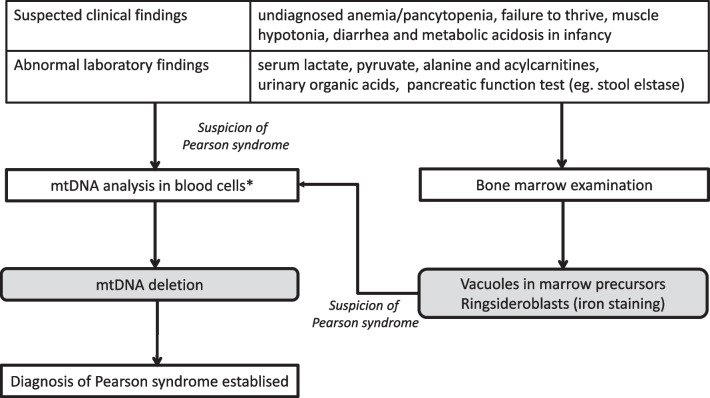
Diagnostic algorithm for suspected Pearson syndrome. Diagnosis of Pearson syndrome is suggested by patient history, clinical symptoms and laboratory findings. The key diagnostic procedures are genetic analysis to detect single large-scale mitochondrial DNA (mtDNA) deletions in blood cells and bone marrow examination. *The single large mtDNA deletion can be also be present in other tissues such as buccal swab and/or urinary epithelial cells in majority of patients [4]

The proposed diagnostic algorithm in PS differs from that of other PMDs (Fig. 3). Metabolic investigations including lactate measurements, urine organic acid analysis and amino acid analysis in plasma can be helpful in the diagnostic work-up. In most cases, typical BM findings with cytoplasmic vacuoles in myeloid and erythroid precursors and ring-sideroblasts will lead to the suspicion of PS. The diagnosis is to be confirmed by mutation analysis of the mitochondrial DNA in peripheral blood. There is a high probability that the single large mtDNA deletion will also be present in other tissues such as buccal swab and/or urinary epithelial cells and the information of the mutational status in these tissues may influence the patient´s management. However, it should be noted that the mtDNA deletion may be detectable only in blood and BM cells in rare cases of PS [4]. Southern blot and long-range polymerase chain reaction analysis can detect SLSMDs, but analysis by next generation sequencing of the entire mitochondrial genome has become increasingly common [39–41]. There is usually no need for a skeletal muscle biopsy with histochemical and biochemical analysis.

### Differential diagnosis

Hematological features of PS, such as pancytopenia, elevated HbF and mean corpuscular volume, marrow hypoplasia and dysplasia resemble those of other inherited BM failure disorders. Diamond- Blackfan anemia (DBA) shares the common feature of hypogenerative anemia in infancy [3, 42], noted in about half of the patients with PS [6]. Patients with Shwachman-Diamond syndrome (SDS) present with exocrine pancreatic insufficiency, failure to thrive, and neutropenia in infancy, features also noted in PS [43]. However, in clinical practice, DBA or SDS can be well distinguished from PS by BM cytology, because vacuolization in marrow precursors and ring-sideroblasts are neither observed in DBA nor SDS. Moreover, most infants with PS have bi- or pancytopenia, while infants with DBA and SDS generally have isolated anemia or neutropenia, respectively. Interestingly, normal reticulocyte counts are frequently reported in patients with PS [3], while DBA patients usually have reticulocytopenia. Erythrocyte adenosine deaminase (eADA) levels are typically elevated in DBA and have been used in the diagnosis of DBA. However, the measurement of eADA is not useful for the differential diagnosis between DBA and PS, because elevated eADA levels were reported in some patients with PS as well [3, 44]. Pediatric hematologists should be aware of the hematological findings in PS, because current standard next generation sequencing panels for congenital anemia or inherited BM failures syndromes usually do not include the analysis of mtDNA. Vacuolization of hematopoietic precursors with or without ring-sideroblasts is a typical finding for PS, but can be seen in other conditions such as copper deficiency, zinc toxicity, riboflavin deficiency, riboflavin transporter deficiency, acute alcoholism, phenylketonuria, chloramphenicol- and linezolid- toxicity as well as other PMDs [45–54].

## Hematological course

### Spontaneous resolution of anemia and clonal evolution

Patients with PS are usually red blood cell transfusion dependent, and often require platelet transfusions during infancy and early childhood as well. The frequency of spontaneous resolution of anemia with transfusion independency was 66% in our German/Austrian study [6]. Hematological recovery usually occurred at the age of 1 to 3 years [6, 7]. Presence of hematological recovery does, however, not imply a better outcome with lower severity of other organ complications [6]. Unfortunately, no predictive factors for spontaneous resolution of anemia have been reported so far. Serial evaluations of heteroplasmy status in peripheral blood cells may possibly be useful to predict hematological remission in the future. Recent reports have suggested that PS patients have a risk of clonal evolution with chromosome 7 aberration similar to other congenital BM failure syndromes: Hoyoux et al. reported on a PS patient who developed a 7q deletion at the age of 2 years [55]. Gagne et al. detected transient monosomy 7 during the course in a child with PS [42]. Nishimura et al. reported a PS patient who developed myelodysplastic syndrome with excess blasts, monosomy 7 and a somatic *RUNX1* mutation in BM cells [56]. Son, et al. reported a patient with PS, who developed myelodysplastic syndrome with excess blasts (13% blasts in BM) at the age of 9 months and received allogeneic HSCT at the age of 10 months [57]. Reynolds et al. reported that 9% of patient with PS developed myeloid malignancies [7]. These reports suggest that PS predisposes to myeloid malignancies.

### Non-hematological complications

**Fig. 4:**
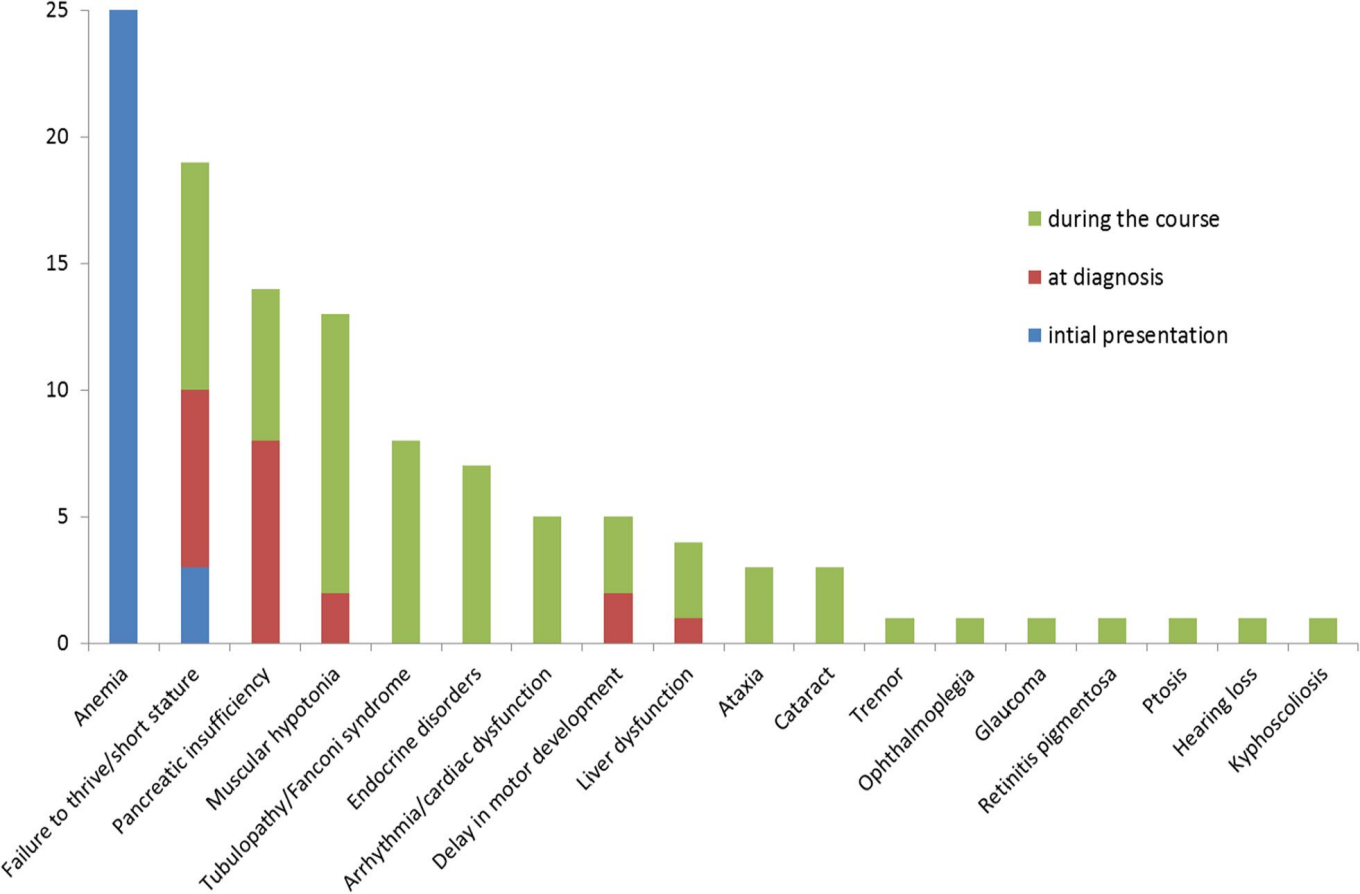
Initial presentation and complications during the clinical course in 25 patients with Pearson syndrome. Signs and symptoms at first presentation (blue), at time of diagnosis (red) or manifesting itself during the clinical course (green) are shown. The left axis provides the number of patients for each item. [6]

Various non-hematological complications generally develop during the course in patients with PS and are generally irreversible. Figure 4 displays complications observed in 25 patients with PS included in our previous retrospective study [6], Table 1 summarizes the incidences and the age at onset for major complications in a most recent review of 139 cases of PS by Ying et al. and 3 largest cohorts of PS in the literatures [5–7, 31]. The majority of patients suffer from failure to thrive resulting in growth retardation and small stature [3–7, 31]. Muscular hypotonia appears early in the clinical course. Dysfunction of the exocrine pancreas is one of the most common non-hematological complications; the incidence ranged from 27 to 62% of patients with PS in the literatures [3, 5–7]. Liver diseases with elevated serum transaminases, bilirubin and/or abnormal sonographic findings are early complications, usually diagnosed before 2 years of age [4–7]. Hepatomegaly is observed in 30–70% of PS patients, while splenomegaly is seen less frequently [3, 7]. Renal involvement with tubulopathy is also common and usually diagnosed at the age of 2–4 years; some patients develop chronic renal failure [3–7]. Endocrine disorders, such as insulin-dependent diabetes, growth hormone deficiency, hypothyroidism, hypoparathyroidism, and adrenal insufficiency can develop at any age [3–7, 44]. Ophthalmological complications including ptosis, retinitis pigmentosa and cataract and cardiac conduction defects are relatively late complications (Table 1) [6, 7]. Some patients have mild delay in motor and speech development in infancy and childhood, while ataxia and tremor appears at a later age [6, 18]. Cognitive impairment is no typical feature of PS. Reynolds et al. reported that 69% of patients had psychiatric problems including anger or rage outbursts, distractibility, and perseveration [6]. Surviving patients typically develop a KSS phenotype (ophthalmoplegia, retinitis pigmentosa, cardiac conduction block and ataxia) [18, 19]. Development of Leigh syndrome has been reported as well [18, 58, 59]. Severe infections are frequent in PS and can result in a fatal course [3, 7].

**Table 1:** Frequency and time of presentation of varied non-hematological complications

	Ying, 2022 (*n* = 139*)	Reynolds, 2021 (*n* = 42**)		Yoshimi, 2021 (*n* = 25)		Rötig, 1995 (*n* = 21)	
Symptoms/organ dysfunctions	%	%	average age	%	median age	%	median age
			(months)		(range, months)		(range, months)
Failure to thrive	49	89	–	72	12 (3–92)	–	–
Muscle hypotonia	12	71	–	52	24 (7–77)	–	–
Pancreatic insufficiency	40	47	20	56	12 (3–31)	57	12 (5–42)
Liver dysfunction	-	66	8	16	18 (10–30)	33	23 (7–36)
Renal disorders	43	80	–	–	–	–	–
(tubulopathy/Fanconi syndrome)	-	37	48	32	41 (21–121)	24	30 (23–45)
Cardiac abnormality/arrhythmia	20	66		20	66 (31–183)	–	–
(cardiac conduction diseases)	–	(17)	(107)	–	–	–	–
Adrenal insufficiency	–	30	57	8	66 (65–67)		
Diabetes mellitus	18	22	111	4	19	10	30 (24–36)
Ophthalmological problem	24	86	–	20	39 (19–92)	–	–
Hearing impairment	3	31	100	4	115		

Data of the most recent review by Ying et al. and 3 largest cohorts of patients with Pearson syndrome (PS) in literatures are displayed (5–7, 31).*The cohort from Ying et al. includes the cases reported by Rötig et al. **The study by Reynolds et al. is based on patient reported outcomes, and the cohort includes 34 cases with PS and 8 pediatric cases with Kearns-Sayre syndrome

### Therapy for patients with Pearson syndrome

Like for the vast majority of PMD patients, there is no cure for children with PS. Therapy is largely supportive to prevent metabolic decompensations during catabolic episodes (eg. fasting, fever, surgery, injury and dehydration) and to treat organ-specific complications [13, 39, 60]. Most PMD patients receive supplements such as coenzyme Q10, L-carnitine and/or various vitamins (vitamin B1, B2, C, E) despite limited evidence for efficacy. Other supportive therapies include sodium bicarbonate substitutions to treat persistent metabolic acidosis, correction of electrolyte abnormalities, fluid infusions and antibiotics. Some medications (eg. valproic acid, statins, and aminoglycosides) should be avoided in PMD because of their effects on mitochondrial function and increased side effects [60]. Patients with PMD also require special attention in case of anesthesia and surgical intervention [39]. We direct the reader to several recent excellent reviews and recommendations of expert panels for management and treatment as well as emerging new therapies of patients with PMDs, because these issues are beyond the scope of this paper [11, 39, 60, 61]. Disease-specific therapies for PS are not available, but awareness of natural history, common complications, and age of their onsets in PS may optimize monitoring and care of patients, and minimize morbidity. Regular monitoring of pancreatic function, renal tubular function, multiple endocrine functions, cardiac function, and follow-up by a multidisciplinary team is necessary to provide optimal care for patients and families (Table 2). PS patients often require pancreatic enzyme replacement and hormone substitution [3, 6]. Tube feeding including placement of percutaneous endoscopic gastrostomy and/or parenteral nutrition is often considered because of severe loss of appetite, dysphagia, feeding difficulty and failure to thrive [7]. Because febrile infections can induce catabolism and trigger metabolic, potentially life-threatening decompensations, the prevention of infections including vaccinations and early antipyretic as well as antibiotic therapy is crucial to minimize the risk. PS patients who developed cardiac conduction defects may require intervention such as ablation and pacemaker implantations [62]. It is recommended that patients with PS carry an emergency protocol, which provides the necessary information for evaluation and optimal care in acute settings.

**Table 2:** Initial and follow-up work-ups for patients with Pearson syndrome

Basic check-ups Physical examination including height/weight, neurological and developmental evaluation CBC with manual differentiation, MCV and reticulocyte count Clinical chemistries with electrolytes including calcium and magnesium, transaminases, BUN, creatinine, glucose, ferritin and phosphate, serum lactate Blood gas analysis Urine analysis for tubulopathy	At diagnosis and every visit
Bone marrow examination with cytogenetic analysis*	At diagnosis and every 12 months until hematological recovery
Endocrine screening tests such as growth chart, pubertal staging, pancreatic function (serum amylase/lipase, stool elastase), thyroid function (TSH, free thyroxine) and hemoglobin A1c	At diagnosis and every 12 months
Cardiac evaluation including echocardiography and ultrasound	At diagnosis and every 12 months
Abdominal ultrasound (pancreas, liver, kidney, spleen)	At diagnosis and every 12 months
Ophthalmologic examination	At diagnosis and every 12 months
Hearing examination	At diagnosis and every 12 months
Provide emergency protocol	

*CBC* Complete blood count, *MCV* Mean corpuscular volume, *BUN* Blood urea nitrogen, *TSH* Thyroid stimulating hormone, *risk of anesthesia should considered

### Therapy of hematological complications

Granulocyte-colony-stimulating factor (G-CSF) may correct neutropenia, but erythropoietin and elthrombopag have limited effect on anemia [6, 63]. Multiple transfusions may cause iron overload in patients with PS. Indication for iron-chelation therapy need to be carefully weighed against the high incidence of spontaneous resolution of anemia, short life expectancy as well as potential toxicities of chelating drugs in PS. Whether iron-chelating therapy in PS could delay progression of organ dysfunctions is unknown.

The indication for hematopoietic stem cell transplantation (HSCT) is often discussed in clinical practice due to persistent transfusion dependency or severe neutropenia. Due to the high rate of spontaneous resolution of anemia, which often occurs in early infancy, it is generally not recommended to consider HSCT before the age of 3–4 years [6, 16]. Although hematological remission can be achieved after HSCT, the potential role of HSCT as therapy for non-hematological complications in PS is unclear. A study in mito-miceΔ carrying mtDNA deletion demonstrated prolonged survival, delayed development of renal failure and suppression of apoptosis of renal cells after BM transplantation [64]. Interestingly, recent studies have demonstrated horizontal mitochondrial transfer between varied cells both in physiological and pathological conditions and mitochondrial transplantation is considered as a potential therapy for varied conditions with mitochondrial dysfunction [12, 65, 66]. The most common donor cells studied for the mitochondrial transcellular transfer are mesenchymal stem cells (MSCs) [65], which can infuse mitochondria into a variety of cells and rescue aerobic respiration of acceptor cells. Moreover, a mitochondrial transfer from donor derived HSCs to damaged recipient MSCs and the metabolic recovery of the recipient MSCs after allogeneic HSCT was recently demonstrated in a mouse model [67]. This study suggests the potential therapeutic role of transplanted HSCs for the recovery of mitochondrial function in some tissues with mitochondrial dysfunction.

Nevertheless, there is no sufficient evidence that HSCT can prolong the survival and correct or prevent the progressive multi-organ dysfunction in patients with PS so far. Moreover, risk for irreversible damage from conditioning with chemotherapy prior to HSCT and greater than expected transplant-related complications should be considered. Six PS patients who received HSCT are reported in the literature [42, 55, 57, 68, 69]. Four of them died following HSCT; one patient died of pulmonary hemorrhage and metabolic acidosis soon after HSCT [57], one patient developed acute myeloid leukemia of recipient origin with 7q deletion and trisomy 8 after second HSCT and died. The causes of death is not reported in two cases. Two patients, who both had an aberration of chromosome 7 prior to HSCT, were alive 3 years and 20 months after cord blood transplantation, respectively. HSCT is currently the only curative treatment for PS patients who developed myeloid malignancy.

### Cell therapy and future direction

There has been great progress in development of novel pharmacological and non-pharmacological treatments including cell and gene therapies for patients with PMDs over the past decade [11, 13, 61]. The first clinical trial of mitochondrial augmentation therapy (MAT) was opened (*ClinicalTrials.gov Identifier: NCT03384420*) and offered patients and families new hope. In this trial, G-CSF mobilized HSCs of PS patients are subjected to ex-vivo mitochondrial augmentation with maternal mitochondria carrying normal mtDNA [9–12]. These autologous HSCs containing normal maternal mtDNA are reinfused in the patient. The advantage of this therapy compared to allogeneic HSCT is that cytotoxic conditioning and transplant-associated complications can be waived. In very preliminary results, improvement of some laboratory findings, neurological complications and quality of life have been described, although how the MAT led to clinical improvement is not fully understood [10]. Further results are awaited to evaluate whether this therapy can prevent or delay the progression of non-hematological complications in PS.

### Prognosis

The prognosis of patients with PS is dismal. About half of patients in the literatures died until the age of 3 years [31] In the German/Austria study including 25 patients with PS, the overall survival at 5 years of age was 58% without demonstrating a plateau [6]. The median age of death was 49 months. Similar survival rates have been reported in other 2 studies [3, 4]. Only few patients with PS have been reported to reach the age of 15 years [5–7, 27], and there is currently no prognostic factor to predict the prognosis of PS patients. The most common cause of death is metabolic crisis with severe and uncontrollable lactic acidosis, followed by infections and multi-organ failures such as liver and kidney failure. Arrhythmia is an important cause of death in older patients.

## Conclusion

A diagnosis of PS needs to be excluded in all infants with hypogenerative anemia. Pediatricians need to be aware of the typical hematological findings, the wide spectrum of complications and a dynamic change in clinical phenotypes during the course in patients with PS. Early diagnosis is crucial for implementation of symptomatic therapy and genetic counseling of families. While curative treatment is currently not available for this dismal disorder, development of cell and gene therapy is expected to offer a new treatment paradigm and hope for affected patients and their families.

## Data Availability

The data of the German/Australian cohort used and/or analysed for this review is published [6] and datasets are available from the corresponding author on reasonable request.

## Abbreviations

PS: Pearson syndrome
mtDNA: Mitochondrial DNA
PMD: Primary mitochondrial disease
SLSMD: Single large-scale mtDNA deletions
CPEO: Chronic progressive external ophthalmoplegia
KSS: Kearns-Sayre syndrome
HSCT: Hematopoietic stem cell
BM: Bone marrow
HbF: Fetal hemoglobin
DBA: Diamond- Blackfan anemia
SDS: Shwachman-Diamond syndrome
G-CSF: Granulocyte-colony-stimulating factor
